# From Cap to Collar: Ontogeny of the Endocytic Collar in *Neurospora crassa*

**DOI:** 10.3390/jof11080577

**Published:** 2025-08-03

**Authors:** Marisela Garduño-Rosales, Caleb Oliver Bedsole, Brian D. Shaw, Rosa R. Mouriño-Pérez

**Affiliations:** 1 Departamento de Microbiología, Centro de Investigación Científica y de Educación Superior de Ensenada (CICESE), Ensenada 22860, B.C., Mexico; 2 The Sainsbury Laboratory, University of East Anglia, Norwich Research Park, Norwich NR4 7UH, UK; marisela.garduno-rosales@tsl.ac.uk; 3 Department of Plant Pathology and Microbiology, Texas A&M University, College Station, TX 77840, USA; olib@tamu.edu (C.O.B.); brian.shaw@ag.tamu.edu (B.D.S.)

**Keywords:** endocytic cap, subapical endocytic collar, endocytosis, exocytosis, *Neurospora crassa*

## Abstract

Endocytosis in filamentous fungi is spatially restricted to a subapical zone known as the endocytic collar, which plays essential roles in membrane recycling and the maintenance of polarized growth. In this study, we investigated the ontogeny of the endocytic collar in *Neurospora crassa* by tracking fimbrin-labeled endocytic patches using confocal microscopy during conidial germination, hyphal branching, and regeneration following mechanical injury. We consistently observed an initial accumulation of endocytic patches at the hyphal tip, forming an apical cap, which later reorganized into a subapical collar. This transition was correlated with a significant increase in elongation rate and the appearance of a Spitzenkörper, indicating a link between exocytosis and collar positioning. Although this correlation is robust, our data do not establish causality; rather, collar formation appears to occur after surpassing a critical elongation. Our findings suggest that exocytosis displaces endocytosis from the apex, resulting in the formation of the collar, which is not required for the establishment of polarized growth but is essential for its maintenance. These results support the development of a unified model of collar formation in filamentous fungi and provide new insight into the spatial coordination between endocytic and exocytic processes during hyphal development.

## 1. Introduction

Endocytosis is a fundamental cellular process that enables eukaryotic cells to internalize plasma membrane components, nutrients, signaling receptors, and extracellular substances. It plays a central role in regulating membrane composition, signal transduction, and maintaining cellular homeostasis [1,2,3,4,5,6,7]. Through endocytosis, cells can respond to environmental changes, regulate receptor signaling, and control membrane tension and surface area [4,8].

Different forms of endocytosis have been described, including clathrin-mediated endocytosis (CME), caveolin-mediated endocytosis, macropinocytosis, and clathrin-independent pathways. Among these, CME is the most studied and involves a complex interplay of cytoskeletal and membrane-associated proteins. CME is the best-characterized and evolutionarily conserved mechanism. CME involves the recruitment of clathrin triskelions to the plasma membrane, which assemble into a polyhedral lattice, driving the formation of coated pits and vesicles [9,10,11,12]. The formation and internalization of clathrin-coated vesicles require a tightly coordinated interplay between adaptor proteins (such as AP2), cargo recognition motifs, phosphoinositides, and dynamic remodeling of the actin cytoskeleton, particularly in organisms under high turgor pressure or mechanical stress, like fungi and plant cells [2,8,13,14,15,16].

Filamentous fungi grow through the extension of tubular hyphae, a highly polarized process that requires a fine-tuned balance between secretion and membrane retrieval [17]. Unlike in many other eukaryotes, endocytosis in filamentous fungi is not distributed across the entire cell surface but is mainly restricted to a defined subapical region [18]. This region, located just behind the apex where active exocytosis occurs, is characterized by a specialized structure known as the endocytic collar. The endocytic collar consists of dynamic patches enriched with key endocytic proteins, including actin, fimbrin, coronin, the Arp2/3 complex, and myosin-1 [19,20,21]. These proteins collectively drive the internalization of plasma membrane domains. The fluorescent dye FM4-64, a well-established marker for endocytosis, is internalized via this pathway, and its uptake is compromised in mutants defective in endocytic proteins. FM4-64 internalization depends on ATP, temperature, and F-actin integrity, reinforcing the idea that this process occurs via active endocytosis [22,23,24,25].

The endocytic collar is thought to serve multiple essential functions. First, it facilitates the recycling of excess membrane generated by intense exocytic activity at the hyphal apex. In *N. crassa*, it has been estimated that approximately 12.5% of the exocytosed membrane is recovered through endocytosis in mature hyphae [26]. Second, the collar contributes to the spatial regulation of cell polarity by recycling components of the apical machinery. The Apical Recycling Model proposes that endocytosis prevents the diffusion of polarity determinants away from the apex, thereby sustaining the focused growth required for hyphal elongation [27,28]. Supporting this model, it has been shown that the distance between the endocytic collar and the hyphal apex is strongly correlated with growth rate in three species of filamentous fungi (*Aspergillus nidulans*, *Colletotrichum graminicola*, and *N. crassa*), suggesting that the precise spatial positioning of the collar plays a more critical role in polarized growth than the absolute rate of endocytosis [29]. These findings reinforce the hypothesis that the spatiotemporal regulation of the endocytic collar is essential for maintaining apical polarity and thus the directional extension of the hyphae.

Although much is known about the molecular composition and functional significance of the endocytic collar, its ontogeny—how it is formed, established, and maintained during development and growth—remains poorly understood. Specifically, previous studies have primarily described the collar in mature hyphae or broadly characterized endocytic protein localization without clearly defining how or why the transition from an apical cap to a subapical collar occurs [27]. It is unclear how endocytic patches are initially organized and what triggers their transition into a structured collar. Moreover, the regulatory mechanisms that coordinate the spatial positioning of endocytosis in relation to exocytosis during dynamic stages such as germination, branching, or regeneration have not been fully elucidated.

Understanding the ontogeny of the endocytic collar is crucial to gaining deeper insights into how filamentous fungi achieve and sustain polarized growth. Dissecting the spatial and temporal dynamics of endocytic patch organization may reveal fundamental principles of membrane trafficking and cytoskeletal coordination in highly polarized cells. Additionally, uncovering the mechanisms behind collar formation could inform broader applications, from fungal biotechnology to antifungal strategies.

In this study, we investigated the ontogeny of the endocytic collar in *N. crassa* using fimbrin tagged with fluorescent proteins (GFP and mChFP) as a live-cell endocytic reporter. We employed confocal microscopy to monitor endocytic patch organization during different stages of hyphal development, including germination, branch formation, and hyphal regeneration following mechanical injury. Our findings shed light on how endocytic patches initially form a cap at the apex and later reorganize into a subapical collar as the hypha reaches a critical elongation rate, explicitly demonstrating for the first time that collar formation is linked directly to surpassing a critical elongation threshold and the emergence of a Spitzenkörper. These observations reveal that while the collar is not essential for the initiation of polarized growth, it is necessary to maintain it, underscoring its importance in the morphogenesis of filamentous fungi.

## 2. Materials and Methods

### 2.1. Strains and Culture Conditions

For all experiments, we used *N. crassa* strains genetically modified to express fluorescently tagged fimbrin, an actin-binding protein involved in endocytosis. The strains employed were TRM08-DD02 (*mat a his-3+::Pccg-1-fim-sgfp+*) and TRM-DD06 (*mat a his-3+::Pccg-1-fim-mchfp+*), which carry single-copy insertions at the *his-3* locus encoding FIM-1 fused to sapphire GFP (sGFP) or monomeric Cherry fluorescent protein (mChFP), respectively, under the control of the constitutive *ccg-1* promoter. These strains allow live visualization of FIM-1 localization and dynamics in germlings and mature hyphae.

Strains were routinely cultured and maintained on Vogel’s Minimal Medium (VMM) supplemented with 1.5% sucrose as a carbon source, under standard laboratory conditions. Inoculations and transfers were performed using sterile techniques, and all handling and propagation of fungal material followed standard genetic and microbiological protocols as described previously [30].

### 2.2. Live-Cell Imaging of Germlings and Mycelia

To study the formation of the subapical endocytic collar during early developmental stages, conidial suspensions of the fimbrin-tagged strains (FIM-1-GFP or FIM-1-mChFP) were prepared at a concentration of 1 × 10^6^ conidia/mL in sterile water. The conidial suspensions were initially incubated at 4 °C for 1 h to ensure synchronized germination, a critical step to observe early polarization events. Following this, the suspensions were evenly spread onto VMM agar plates and incubated at 30 °C for 4 to 9 h, allowing the formation and elongation of germ tubes.

For live-cell microscopy, we employed the “inverted agar block” technique [31]. Small agar blocks containing germlings were excised, inverted onto a glass slide, and gently sealed with a coverslip. This method ensures minimal sample movement and optimal imaging conditions.

Imaging was conducted using an Olympus FV1000 FluoView™ laser scanning confocal microscope (Olympus, Tokyo, Japan) equipped with a UPlanFLN 60× (NA 1.42) oil immersion objective (Olympus, Tokyo, Japan). This system allows high-resolution, live imaging of dynamic cellular processes. Fluorescence and Differential Interference Contrast (DIC) images were acquired simultaneously using FluoView™ software version [4.2] (Olympus, Tokyo, Japan), enabling correlation of protein localization with hyphal morphology. To ensure consistency in the classification of endocytic patch organization, we established operational criteria to distinguish between apical caps and subapical collars. Cap-like patterns were defined as concentrated clusters of endocytic patches located within 1 µm of the hyphal apex, forming a compact, tip-focused domain. In contrast, collars were identified as circumferential rings of discrete patches positioned 1.5–3.5 µm behind the apex. These morphological and positional parameters were applied uniformly across all datasets to minimize subjectivity and enhance the reproducibility of the analysis.

To analyze collar formation in lateral branches of mature hyphae, mycelial plugs of *N. crassa* FIM-1-GFP were incubated on VMM plates at 30 °C for 16–18 h, promoting robust mycelial expansion and branching. Again, the inverted agar block method was used for sample preparation. Imaging in this case was performed on a more advanced Olympus FV3000 FluoView™ confocal system integrated with an Olympus IX83 inverted microscope (Olympus, Tokyo, Japan), featuring a Galvanometer scanner and High Sensitivity GaAsP PMT detectors for improved signal detection. Two objectives were used for different magnifications: the UPlanSApo 40× (NA 1.25) (Olympus, Tokyo, Japan) with silicone oil immersion and the UApo N 100× OTIRF (NA 1.49) (Olympus, Tokyo, Japan) with standard oil immersion (type-F). Z-stack time-lapse acquisitions were captured simultaneously in fluorescence and bright-field channels to monitor collar formation in three dimensions over time.

### 2.3. Live-Cell Imaging of Regenerating Hyphae Following Mechanical Injury

To investigate the reorganization of endocytic structures during hyphal regeneration, we mechanically injured mature hyphae expressing FIM-1-GFP. Mycelia were grown from mycelial plugs on VMM plates until they reached the colony edge. At this point, hyphae at the margin were manually cut using a sterile scalpel, simulating mechanical damage.

Following injury, the area was immediately located using bright-field microscopy. Time-lapse imaging was then initiated to follow FIM-1 dynamics in hyphal compartments located just ahead of the wound site, capturing the temporal and spatial changes associated with the regeneration response. Imaging was performed for 2–3 h post-injury to monitor the entire process from damage to regrowth.

This analysis was carried out on the Olympus FV1000 FluoView™ confocal microscope system (Olympus, Tokyo, Japan) using a UPlanFLN 60× (NA 1.42) oil immersion objective (Olympus, Tokyo, Japan). Fluorescence and DIC images were recorded simultaneously to track protein localization relative to morphological changes. Acquisition was controlled using FluoView™ software version [4.2] (Olympus, Tokyo, Japan).

### 2.4. Image Processing

All acquired images and time-lapse series were post-processed and prepared for presentation using Adobe Photoshop 2022 (Adobe Systems Inc., San Jose, CA, USA). Final image adjustments were limited to cropping, contrast enhancement, and assembly into figures. All brightness and contrast modifications were linear and applied equally across all samples. No nonlinear image processing was applied.

### 2.5. Statistical Analysis

All statistical analyses were performed using STATISTICA version [9.0] data analysis software system (TIBCO Software Inc.), and Graphs were made with GraphPad Prism version [9.0] (GraphPad Software, San Diego, CA, USA). To compare hyphal elongation rates between developmental stages characterized by an apical cap or a subapical collar, data normality was first assessed using the Shapiro–Wilk test. For comparisons between two independent groups, such as germlings displaying a cap (*n* = 25) versus a collar (*n* = 14), the non-parametric Mann–Whitney U test was used. For paired comparisons within the same hypha, such as elongation rates in branches (*n* = 17) and regenerating hyphae (*n* = 11) before and after collar formation, paired *t*-tests were applied.

Additionally, linear regression analyses were conducted to evaluate the relationship between time and hyphal elongation during branch formation, with separate regression models fitted for the cap and collar stages. Confidence intervals (95% CI) were calculated for all regression lines, and differences were considered statistically significant when p-values were less than 0.05. All data are presented as mean ± standard error of the mean.

## 3. Results

To investigate the ontogeny of the endocytic collar in *N. crassa*, we analyzed the dynamics of endocytic patches labeled with FIM-1-GFP or FIM-1-mChFP using laser scanning confocal microscopy. We focused on three distinct developmental contexts: conidial germination, hyphal branching, and hyphal regeneration after mechanical injury. In all cases, we observed a consistent transition from an apical cap-like structure to a subapical collar correlated with increased elongation rate.

### 3.1. Endocytic Patches Form a Cap During Germling Development

**Figure 1:**
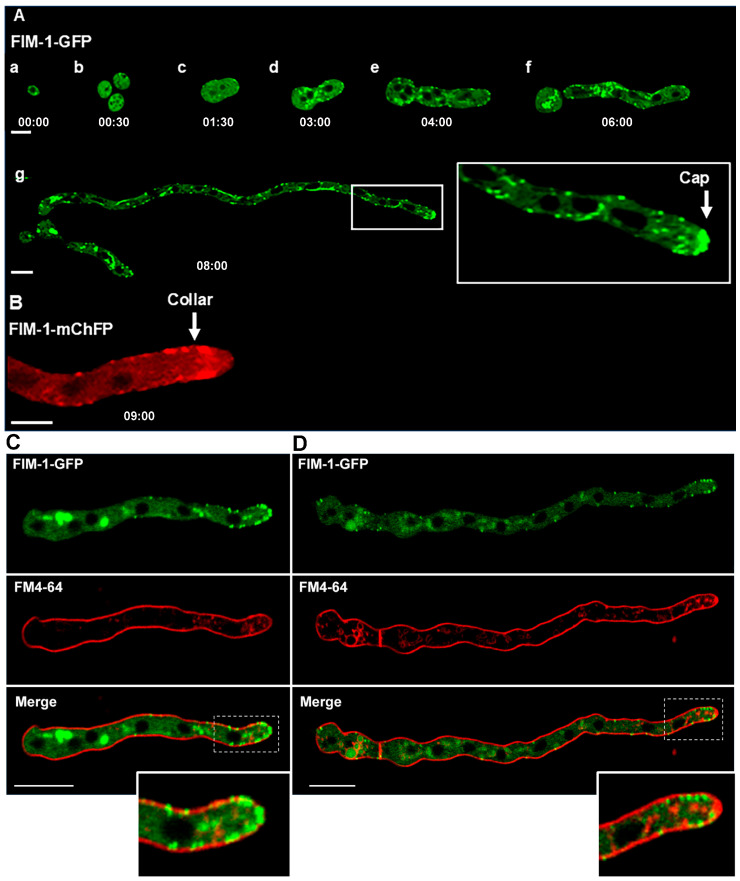
Fimbrin localization during conidial germination in *Neurospora crassa*. (**A**) Conidia and germlings tagged with FIM-1-GFP; (**a**–**d**) Patches localize throughout the plasma membrane in conidia and germlings. (**e**,**f**) Additionally, patches begin to accumulate at hyphal tips in germlings. Scale bar = 5 µm. (**g**) As the tube grows, apical patches organize into a cap, as shown in the apex zoom-in. Other patches are visible along the tube. Scale bar = 10 µm. (**B**) FIM-1-mChFP; 9 hpi (300 µm long) germling displays a subapical collar arrangement of patches (arrow). Scale Bar = 10 µm. (**C**) Fimbrin localization and Spitzenkörper presence during germling development in germling at 6 h post-inoculation (hpi), showing an apical cap of Fimbrin-labeled patches and no visible Spitzenkörper. (**D**) Germling at 7 hpi displaying a subapical collar and a clearly defined Spitzenkörper. Scale Bar = 10 µm.

Using strains expressing FIM-1-GFP or FIM-1-mChFP, we monitored conidial germination and the establishment of the endocytic collar (Figure 1). In dormant conidia, patches were dispersed throughout the plasma membrane. Upon germ tube emergence, patches accumulated at the site of outgrowth (Figure 1A(a,b)). As the germ tube elongated, endocytic patches became concentrated at the apex, forming a cap-like structure (Figure 1A(g)). Additional patches remained visible along the germ tube.

**Figure 2:**
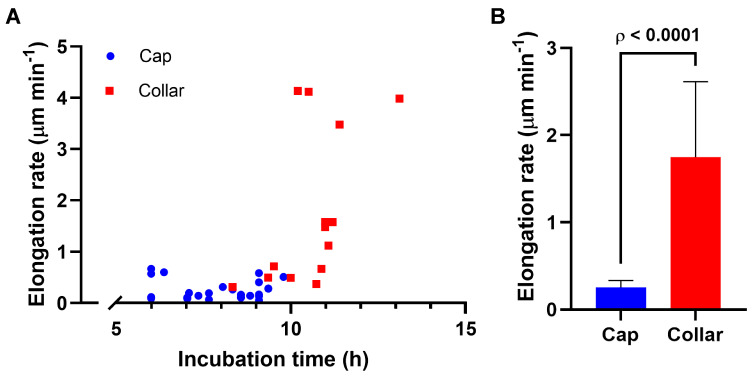
Comparison of the elongation rate in germlings with a cap or collar. (**A**) Scatter plot showing the instantaneous elongation rate (µm min^−1^) of individual germlings (*n* = 39) measured at a specific time point during incubation. Each point corresponds to a single germling displaying either an apical cap (blue) or a subapical collar (red), allowing comparison of elongation behavior across developmental stages. The x-axis indicates the time post-inoculation at which the measurement was taken. (**B**) Column chart showing the average elongation rate (µm min^−1^) in germlings displaying either a cap (*n* = 25) or a collar (*n* = 14). Error bars represent ±95% CI. ρ < 0.0001 (two-tailed, Mann–Whitney test).

Once the germ tube reached approximately 150 µm in length, the cap-like structure shifted to a subapical position, forming a well-defined endocytic collar (Figure 1B). Elongation rates were significantly higher in germlings with a collar compared to those with a cap (1.75 ± 0.4 µm min^−1^ vs. 0.26 ± 0.04 µm min^−1^; *n* = 14 and 25, respectively; Figure 2 and Table 1). Growth rate was 6.83-fold higher in germlings with endocytic patches forming an actual collar. Notably, germlings with an apical cap did not display a visible Spitzenkörper with FM4-64 staining, whereas those with a subapical collar did (Figure 1C,D).

### 3.2. Transition from Cap to Collar During Branch Formation

**Figure 3:**
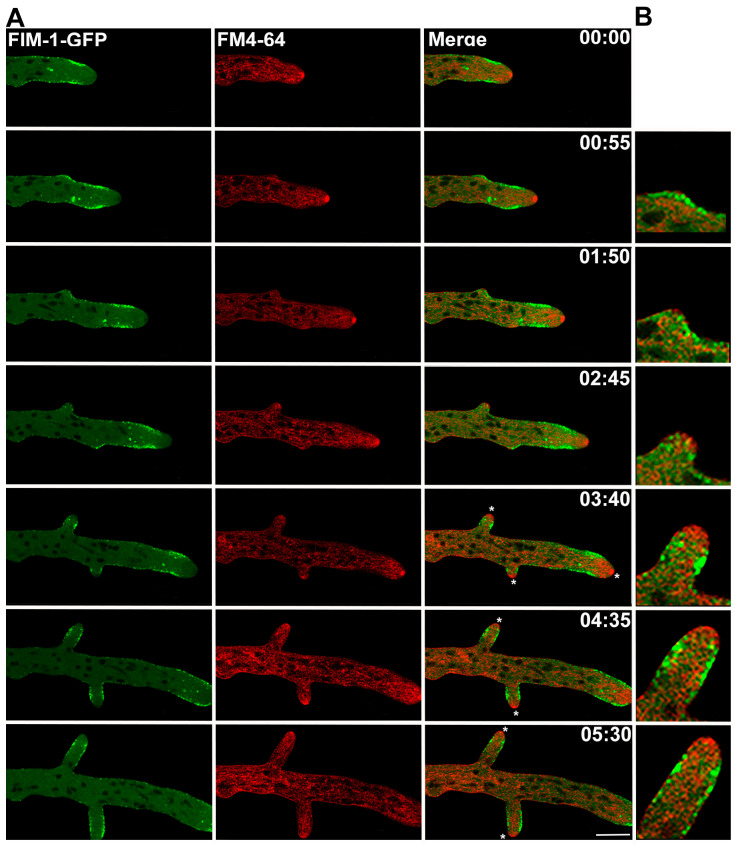
Fimbrin dynamics, Spitzenkörper localization, and cap-to-collar transition during branch formation in *Neurospora crassa*. (**A**) Time-lapse images showing the formation of a lateral branch in a hypha expressing FIM-1-GFP and stained with FM4-64 to follow the Spitzenkörper formation. The asterisk indicates the appearance of the Spitzenkörper associated with collar formation. Arrows point to the cap, and the arrowheads to the collar. (**B**) shows a magnified view (zoom-in) of the branch apex, highlighting the spatial relationship between the endocytic structures and the Spitzenkörper. Scale bar = 10 µm. Time = mm:ss.

We next examined whether a similar cap-to-collar transition occurred during lateral branch formation. In young branches of hyphae expressing FIM-1-GFP, endocytic patches initially accumulated at the lateral site of the emerging protuberance (Figure 3). As the branch elongated, patches organized into an apical cap, which subsequently transitioned to a subapical collar as elongation continued (Figure 3).

**Figure 4:**
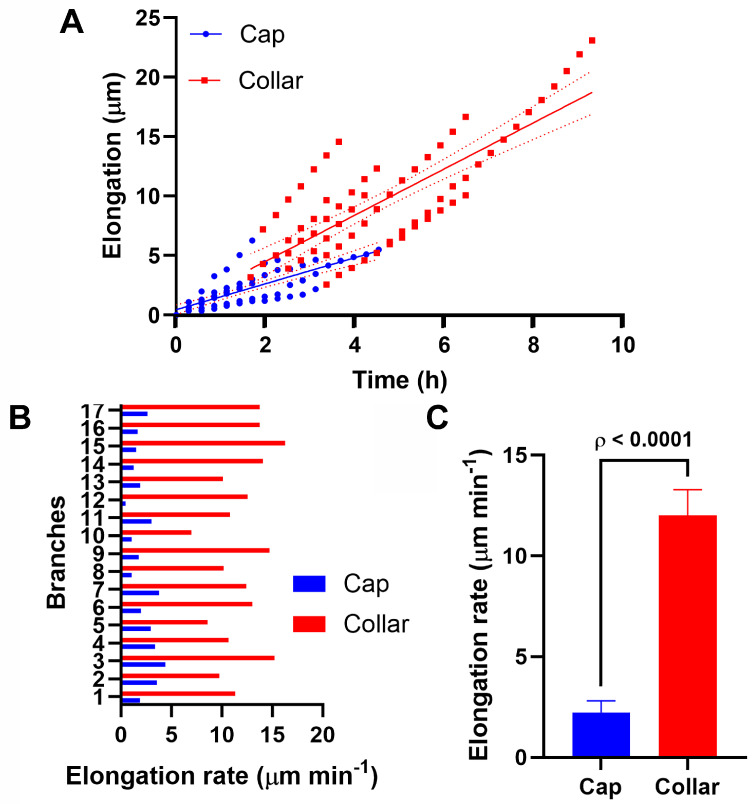
Comparison of the elongation rate in emerging branches with a cap (blue) or collar (red). (**A**) Scatter plot showing new branches’ time points having either a cap (*n* = 70) or collar (*n* = 70) and their elongation (µm). Cap y = 1.09x + 0.45 (ρ < 0.0001); collar y = 1.94x + 0.60 (ρ < 0.0001). Regression analysis line ± 95% CI. (**B**) Bar chart showing the comparison of new branches’ elongation rate (µm min^−1^) while having a cap and a collar (*n* = 17). Each number on the y-axis corresponds to the same branch for a cap and collar. Data are means. (**C**) Column chart showing the average elongation rate (µm min^−1^) in new branches displaying cap and collar. Error bars are ± 95% CI. ρ < 0.0001 (two-tailed, paired *t*-test).

Quantitative analysis showed that elongation rate increased significantly when the collar was established (12.00 ± 0.60 µm min^−1^ vs. 2.23 ± 0.26 µm min^−1^ in branches with collar vs. cap; *n* = 17; Figure 4, Table 1). Additionally, a regression analysis revealed that the collar’s presence was strongly correlated with this shift in growth dynamics, exhibiting a steeper linear relationship with growth rate (y = x1.6471, R^2^ = 0.765) compared to the cap (y = x1.008, R^2^ = 0.6095). These results suggest a functional transition in endocytic organization, where the cap may represent an initial or preparatory stage, and the collar a final destination of endocytic patches supported by rapid polarized growth during active branch extension.

### 3.3. Collar Formation in Regenerating Hyphae After Injury

**Figure 5:**
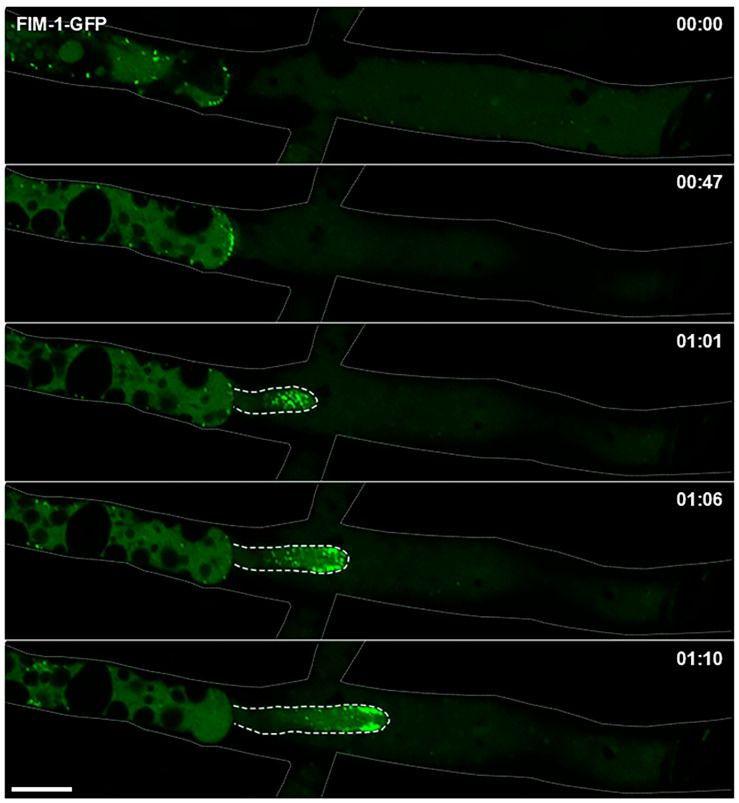
Regenerating hypha following mechanical injury of *Neurospora crassa* tagged with FIM-1-GFP. Time-lapse shows the regeneration of an injured hypha, with a new tip emerging from the plugged septal pore. Patches accumulate at the plugged septum forming a “crescent”-shaped structure, then the new tip emerges, and patches organize as a cap the later transitioning into a collar. The thin line indicates the profile of the injured hyphae, while the dotted line shows the profile of the regenerating hyphal growing inside the injured one. Scale Bar = 10 µm. Time = hh:mm.

To assess collar formation during regeneration, we analyzed hyphae expressing FIM-1-GFP following mechanical injury. Endocytic patches first accumulated at the plugged septum of the severed hypha, forming a crescent-shaped cluster. Subsequently, a new tip emerged, with endocytic patches organizing into an apical cap (Figure 5). Approximately 60 min after injury, the patches relocalized into a subapical collar.

**Figure 6:**
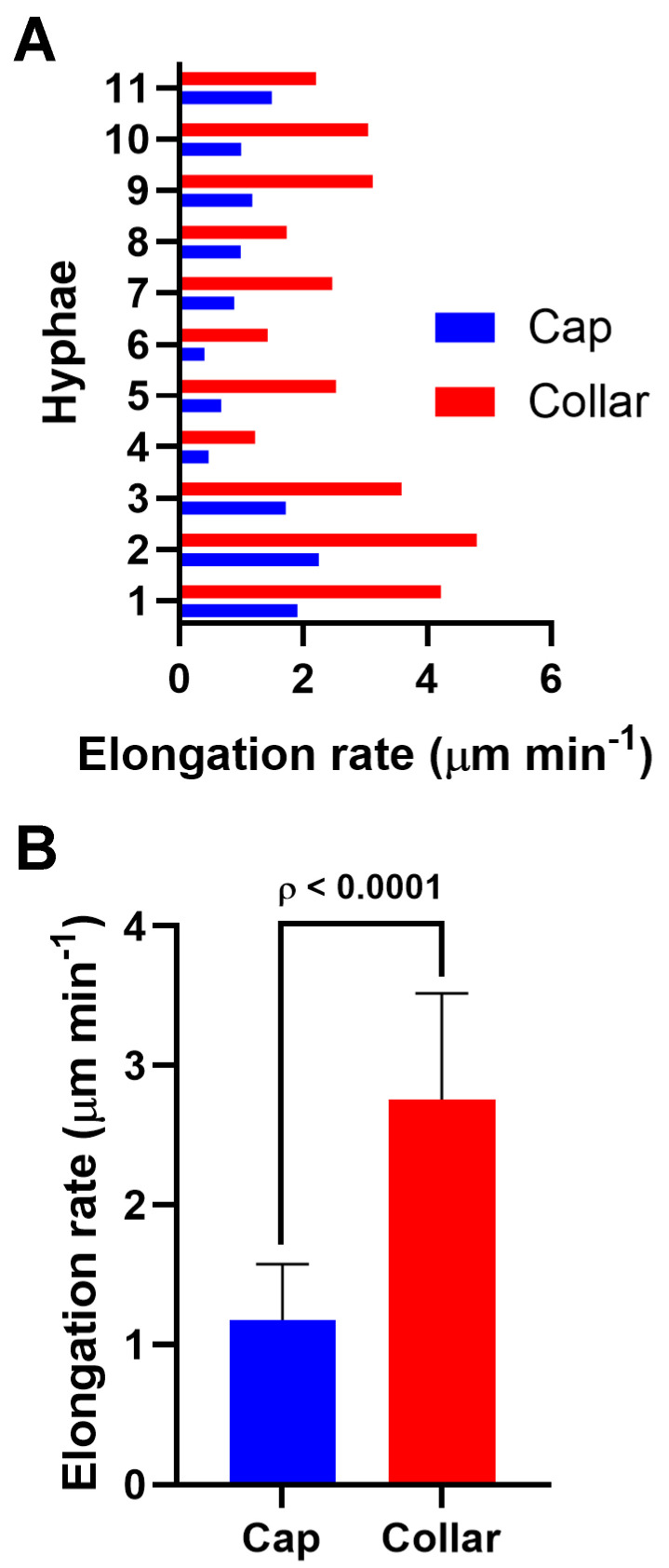
Comparison of the elongation rate in emerging hyphae after mechanical injury with a cap or collar. (**A**) Bar chart showing the comparison of regenerating hyphae’s elongation rate (µm min^−1^) while having a cap (blue) and a collar (red) (*n* = 11). Each number on the y-axis corresponds to the same hypha for a cap and collar. Data are means. (**B**) Column chart showing the average elongation rate (µm min^−1^) in regenerating hyphae displaying cap and collar. Error bars are ± 95% CI. ρ < 0.0001 (two-tailed, paired *t*-test).

The elongation rate measurements confirmed a higher rate when patches were organized as a collar compared to a cap (2.75 ± 0.34 µm min^−1^ vs. 1.17 ± 0.18 µm min^−1^; *n* = 11; Figure 6, Table 1). These results are consistent with those from germlings and branches, supporting the idea that collar formation is triggered once a critical elongation rate is reached.

In all developmental contexts analyzed, we observed a conserved sequence in the organization of endocytic patches: initial dispersion, accumulation into an apical cap, and eventual formation of a subapical collar. This transition consistently coincided with a marked increase in elongation rate and the emergence of a visible Spitzenkörper. These findings support the model that exocytosis-driven elongation displaces endocytosis to the subapex, resulting in the formation of the endocytic collar.

## 4. Discussion

This study provides the first detailed analysis of the ontogeny of the endocytic collar in *N. crassa*, demonstrating a consistent transition from apical cap-like structures to subapical collars during conidial germination, hyphal branching, and regeneration following injury. In all cases, collar formation was associated with a significant increase in hyphal elongation rate and coincided with the emergence of a visible Spitzenkörper.

Our findings suggest that the initial cap-like accumulation of endocytic patches marks a preparatory phase for polarized growth. This apical arrangement of patches is gradually displaced to the subapical region once exocytic activity becomes dominant, forming the mature endocytic collar. The correlation between collar formation and elongation rate supports the idea that exocytosis physically and functionally restricts the localization of endocytosis to the subapex [25,28,32]. Although our observations reveal a strong correlation between collar formation and the surpassing of a critical elongation threshold, they do not demonstrate causality. Thus, while our data are consistent with a model in which exocytosis drives the spatial reorganization of endocytic activity, this remains a hypothesis that was not directly tested in this study. We did not label exocytic machinery or vesicle flux and therefore cannot confirm mechanistic displacement. Future experiments using markers for exocytic components such as Rab GTPases or exocyst subunits will be necessary to directly validate this model. This idea is aligned with the Apical Recycling Model proposed by [27], which suggests that endocytosis at the subapex plays a key role in recycling the polarity machinery and sustaining directional growth.

Previous work has described the localization and importance of endocytic proteins such as fimbrin, coronin, and MYO-1 in *N. crassa* [19,20,21] and *A. nidulans* [33]. Our study complements these findings by showing the dynamic spatial reorganization of these proteins during early hyphal development. The observation that germlings with a cap structure did not show a Spitzenkörper when stained with FM4-64, while those with a collar did, aligns with previous reports linking Spitzenkörper visibility to a critical stage in hyphal growth [34].

**Figure 7:**
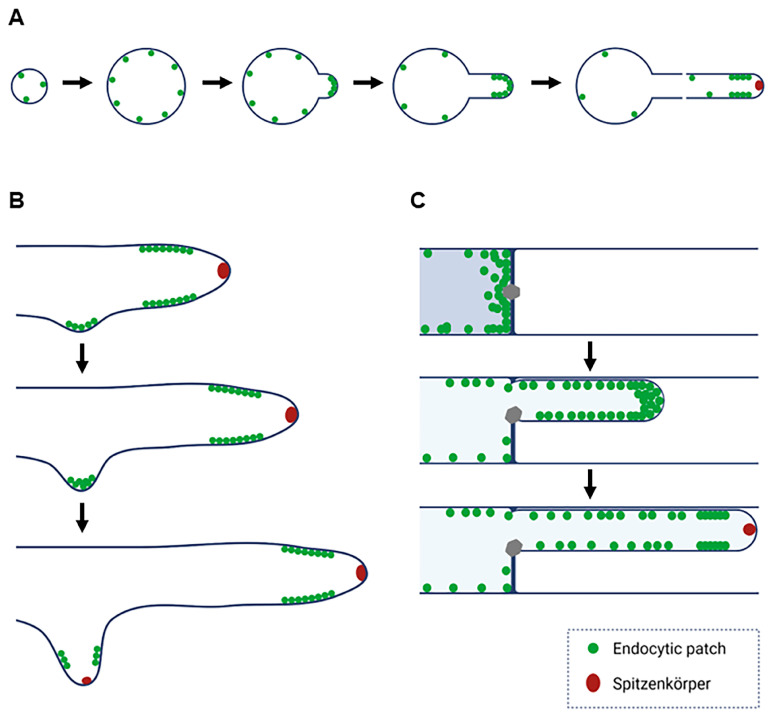
Models “From cap to collar”. (**A**) Depicts the distribution and accumulation of endocytic patches through time during germination and germling growth. Initially, a few patches are scattered throughout the conidium, and once they swell, the patches become more numerous. Some patches are then visible at the site of tip emergence, and as the tube elongates, patches arrange in a “cap”-like structure. Then, there is a switch from a cap (apex) to a collar (subapex) due to higher exocytosis. (**B**) In a similar way, endocytic patches accumulate at sites where branches are about to emerge, then they organize into a cap that transitions to a collar shortly after. (**C**) In regenerating hyphae, we observe a similar phenomenon, where endocytic patches accumulate in a crescent at plugged septa, where the new tips emerge. Patches form caps in these regenerated hyphae and then transition to collars. In all cases, when the patches form the collar, we also observe the Spitzenkörper.

The conservation of this cap-to-collar transition across germlings, branches, and regenerating hyphae points to a shared underlying mechanism (Figure 7). Based on recent evidence, we propose that a critical elongation rate—likely driven by increased exocytic activity—triggers the spatial reorganization of endocytic patches, leading to their relocation from the apex to a defined subapical zone. This shift is consistent with findings showing that the distance between the endocytic collar and the hyphal apex closely correlates with the hyphal growth rate. These observations highlight the importance of collar positioning, rather than the rate of endocytosis itself, as a key factor in maintaining apical polarity during rapid hyphal elongation [33]. This displacement may prevent interference between exocytic and endocytic processes at the apex, ensuring efficient membrane recycling and polarized growth. Although our results clearly demonstrate that collar formation coincides with critical changes in elongation rate and Spitzenkörper emergence, the molecular triggers for this transition remain unknown. Several candidates merit further investigation. First, BAR-domain proteins, known to sense and induce membrane curvature, could respond directly to increased exocytic activity at the apex by reorganizing endocytic patches into a collar structure positioned to efficiently manage membrane recycling [35,36,37,38]. Second, Rho-family GTPases (e.g., Cdc42 or Rac homologs) represent likely regulators, as they coordinate cytoskeletal dynamics and polarized growth in fungi, potentially linking growth signals to the spatial reorganization of endocytic machinery [39,40,41]. Third, intracellular calcium gradients are compelling candidates given their established roles in regulating polarized growth and vesicle trafficking in fungal hyphae; changes in local calcium concentrations at the growing apex might act as signals to initiate the cap-to-collar transition [42,43].

In addition to these possibilities, other mechanisms could contribute to the spatial repositioning of endocytosis. The local increase in exocytic vesicle accumulation during rapid growth could generate physical crowding at the apex, displacing endocytic sites toward the subapical region. Membrane tension, dynamically modulated by exocytosis and tip expansion, may act as a mechanical signal to trigger this transition [44]. Moreover, the redistribution of phosphoinositides, such as PI(4,5)P_2_, at the plasma membrane during intense exocytic activity might influence the recruitment and stabilization of endocytic adaptors [11,12].

Recent studies suggest that cytoskeletal rearrangements, particularly actin dynamics, may not only respond to growth signals but also actively participate in feedback mechanisms that regulate endocytic organization during polarized growth. In *N. crassa* and other filamentous fungi, actin-binding proteins such as fimbrin, coronin, and myosin I are essential for stabilizing endocytic patches at the subapex [19,20,21]. Disruption of actin nucleators or associated proteins leads to defective endocytosis and loss of polarity, indicating that proper actin network formation is critical for maintaining the spatial separation between exocytic and endocytic domains [13,33]. Moreover, actin polymerization has been shown to reinforce endocytic site maturation and vesicle internalization, suggesting a dynamic coupling between membrane trafficking and cytoskeletal architecture [2,4]. This feedback likely ensures that as hyphal elongation intensifies and membrane demands increase, endocytic machinery can adapt spatially to sustain efficient recycling and growth. Thus, collar formation may not simply be a passive consequence of increased exocytosis, but rather the outcome of an integrated system in which actin dynamics and membrane trafficking co-regulate each other to maintain hyphal morphogenesis. Together, these speculative models highlight the complexity of the mechanisms regulating membrane trafficking during hyphal development and suggest that collar formation may integrate multiple mechanical and biochemical cues.

Our results are also in line with previous estimates that approximately 12.5% of exocytosed membrane is recycled via the endocytic collar in mature hyphae of *N. crassa* [26]. The strategic subapical positioning of the collar likely allows efficient membrane retrieval without disrupting the exocytic flow at the tip.

Although our study focuses on the spatial dynamics of endocytosis, it raises new questions regarding the molecular signals that couple growth rate to collar formation. Future studies could explore whether components of the cytoskeleton, signaling pathways, or mechanical feedback mechanisms are involved in sensing growth rate and repositioning endocytic activity.

These findings open several avenues for future investigation. First, although fimbrin is a reliable marker of endocytic patches in filamentous fungi, complementary imaging with additional endocytic components such as coronin or actin regulators could provide further insight into collar maturation. Moreover, the proposed displacement of endocytosis by exocytosis remains hypothetical; future experiments using fluorescently tagged exocytic machinery (e.g., Rab GTPases or exocyst subunits) will be necessary to test this model directly. It would also be valuable to explore the molecular signals—such as calcium gradients, BAR-domain proteins, or Rho-family GTPases—that may coordinate the spatial reorganization of endocytic domains in response to growth rate. Finally, the use of mutants lacking key endocytic proteins or cytoskeletal regulators could clarify the functional contribution of the collar to hyphal morphogenesis and polarity maintenance.

In conclusion, this study supports a model in which the transition from an apical cap to a subapical collar is a conserved and regulated process in *N. crassa* development. Collar formation appears to be essential for the maintenance—but not the initiation—of polarized growth and is likely governed by the balance between endocytic and exocytic activity at the hyphal tip.

## Figures and Tables

**Table 1:** Comparison of hyphal elongation rates during cap and collar stages in different developmental contexts of *Neurospora crassa*.

Developmental Stage	Patches Organization	Elongation Rate (µm/min)	Elongation Ratio
Germling	Cap	0.26 ± 0.04	6.83
	Collar	1.75 ± 0.4	
Branch	Cap	2.23 ± 0.26	5.38
	Collar	12.00 ± 0.60	
Hyphal Regeneration	Cap	1.17 ± 0.18	1.58
	Collar	2.75 ± 0.34	

## Data Availability

The original contributions presented in this study are included in the article. Further inquiries can be directed to the corresponding author.
